# Hemoglobin and total bilirubin may affect tacrolimus clearance in liver transplant patient following the early postoperative period: a case report

**DOI:** 10.1186/s13256-024-04753-3

**Published:** 2024-09-05

**Authors:** Vichapat Tharanon, Pansa Iamrahong, Jutamas Tuamsem, Kunvadee Choochaeam, Titinun Auamnoy, Abhasnee Sobhonslidsuk

**Affiliations:** 1 Clinical Pharmacy section, Department of Pharmacy, Faculty of Medicine, Ramathibodi Hospital, Mahidol University, Bangkok, Thailand; 2 Division of Social Pharmacy and Pharmacy Administration, Faculty of Pharmaceutical Sciences, Burapha University, ChonBuri, Thailand; 3 Division of Gastroenterology and Hepatology, Department of Medicine, Faculty of Medicine, Ramathibodi Hospital, Mahidol University, Bangkok, Thailand

**Keywords:** Hemoglobin, Total bilirubin, Tacrolimus, Liver transplant patient, Early postoperative period, Case report

## Abstract

**Background:**

Tacrolimus is a potent calcineurin inhibitor (CNI) that is principally used as a first-line immunosuppressant for the prophylaxis of allograft rejection in liver transplantation (LT) patients. In clinical practice, prescribing the optimal tacrolimus dosage is complicated by its narrow therapeutic index and high pharmacokinetic variability. Thus, performing therapeutic drug monitoring (TDM) of only tacrolimus may not provide optimal drug levels. However, other influential clinical factors affecting tacrolimus levels, such as hemoglobin (Hb), hematocrit, and total bilirubin (TBIL), should be considered while adjusting tacrolimus levels. This case report aims to introduce clinicians and their teams to taking the pharmacokinetic prediction equation into consideration for a better understanding of tacrolimus dosage adjustment during the early postoperative LT.

**Case presentation:**

In this case report, an 18-year-old male patient of Thai ethnicity was admitted for orthotropic liver transplantation, and tacrolimus was prescribed as a cornerstone immunosuppressive agent. In the immediate postoperative period, which is the most challenging period in liver transplantation, the population pharmacokinetics predictive equation was clinically used to assist in dosage adjustment of tacrolimus by considering the significant clinical factors in this case.

Hemoglobin and total bilirubin levels were deemed significant clinical factors affecting the oral clearance (CL/F) of tacrolimus. First, a decrease in the Hb concentration increases the free drug concentration and therefore increases the CL/F of tacrolimus. Second, an elevated TBIL decreases the biliary excretion of tacrolimus, resulting in a decrease in the CL/F of tacrolimus. Thus, dose optimization of tacrolimus would be accurate when taking the pharmacokinetic prediction equation into consideration. Moreover, the results may contribute to a better understanding of tacrolimus pharmacokinetic variability in each transplant patient during the immediate postoperative course.

**Conclusions:**

Hemoglobin and total bilirubin were significant clinical factors influencing the oral clearance of tacrolimus early after liver transplantation. A decrease in the hemoglobin concentration would increase the free drug concentration and therefore increase the oral clearance of tacrolimus. An elevated total bilirubin decreases the biliary excretion of tacrolimus, resulting in a decrease in the oral clearance of tacrolimus.

## Background

Tacrolimus, a potent calcineurin inhibitor (CNI), is principally used as an immunosuppressant for the prophylaxis of allograft rejection in various types of organ transplantations. It works by targeting T-cell activation through the inhibition of calcineurin phosphatase, a key enzyme in T-cell receptor signaling and cytokine production. The European Association for the Study of the Liver (EASL) recommended tacrolimus as the first-line treatment for liver transplantation (LT) patients on the basis of the best evidence of post-transplant reduction in mortality, graft loss, graft rejection, and steroid-resistant rejection [1, 2]. The establishment of optimal postoperative tacrolimus dosing is beneficial for improving the clinical outcomes of LT patients. Monitoring of the trough tacrolimus concentration is a routine practice in clinical settings to optimize tacrolimus treatment and minimize adverse effects [3]. Prescribing the optimal tacrolimus dose is complicated by its narrow therapeutic index, high pharmacokinetic variability, and the impact of genetic factors [4]. However, using only routine therapeutic drug monitoring (TDM) may not provide the optimal dose of tacrolimus because a number of clinical factors affect tacrolimus pharmacokinetics. The reported clinical factors influencing tacrolimus pharmacokinetics in LT patients included weight, hemoglobin, age, sex, ethnicity, time post transplantation, type of transplanted organ, hepatic function, food, drug interaction, *CYP3A5* polymorphisms, and the inherent complexity of the clinical condition [5–7]. Therefore, applying the pharmacokinetic prediction equation together with routine TDM in practice might be more successful in assisting in designing and individualizing the critical tacrolimus dosage for achieving the target tacrolimus concentrations in the early period post-LT [8]. Moreover, an understanding of the tacrolimus pharmacokinetic variability of each transplant patients could be better described.

## Case presentation

An 18-year-old Thai man who weighed 70 kg (kg) and was 1.65 m tall (body mass index 25.71 kg/m^2^) was admitted to the organ transplantation unit for orthotropic liver transplantation (OLT). He was diagnosed with biliary atresia post-Kasai operation at 3 months of age with biliary cirrhosis and portal hypertension. The model for end-stage liver disease (MELD) score was 17, and the Child–Turcotte–Pugh classification (score) was B [9]. Transplantation was performed on 9 March 2022. The cold ischemia time was 7 hours and 7 minutes, and the warm ischemia time was 1 hours and 35 minutes. He successfully underwent OLT from a 47-year-old male donor. Immediately after transplantation, the patient was transferred to the intensive care unit (ICU) for postoperative care. During ICU admission, he developed septic shock from intra-abdominal infection with suspected bacterial translocation and acute kidney injury from massive blood loss and hypotension. He was then resuscitated with adequate fluid replacement, norepinephrine (0.09–0.29 mcg/kg/min), and an empirical antibiotic (imipenem) to improve hemodynamic instability and infection treatment. He did not develop ascites or pleural effusion postoperatively. The vasopressor was discontinued on postoperative day (POD) 3 with improved clinical symptoms. He received standard immunosuppressive treatment with mycophenolate mofetil (Cellcept^®^ 500 mg; F. Hoffmann-La Roche Ltd., Basel, Switzerland) and corticosteroids (intravenous methylprednisolone sodium succinate; Solu-medrol^®^; Pfizer Manufacturing Belgium NV, Puurs, Belgium) on PODs 1–6 and then was switched to oral prednisolone on POD 7 and onward. Oral immediate-release tacrolimus (Prograf^®^ 0.5 and 1 mg; Astellas, Kerry, Ireland) was carefully prescribed every 12 hours at the same time each day (7 a.m. and 7 p.m.) to avoid food and maintain the blood concentration of tacrolimus. The first dose of tacrolimus was initially given at 1 mg every 12 hour on POD 3, and the subsequent tacrolimus doses were adjusted to maintain tacrolimus concentrations within the target range (5–7 ng/mL) without adverse effects. Furthermore, the other oral prescribed medications included omeprazole, trimethoprim/sulfamethoxazole, acyclovir, and ursodeoxycholic acid. Within a few days, the patient gradually recovered from septic shock and completed 7 days of imipenem treatment. Then, he was transferred back to the organ transplant unit on POD 7 for the best postoperative observation, immunosuppression with allograft function monitoring, and medication-related education.

The TDM of trough tacrolimus concentrations was monitored at 6.30 a.m. before administering the tacrolimus morning dose every day. During his hospitalization, his trough tacrolimus concentrations and essential laboratory tests were routinely evaluated according to the post-LT protocol. The trough tacrolimus concentrations were highly variable during the first 14 PODs; concentrations ranged from 3.5 to 12.2 ng/mL from a dose of 1–7 mg/day. There are a few days (POD 8–10) in which tacrolimus concentrations were within the therapeutic range. Thus, the daily tacrolimus doses were adjusted to achieve the therapeutic target during the early post-LT period. On POD 15, the tacrolimus trough concentration was monitored before administering 1 mg of tacrolimus; the trough concentration was reported to be too high (12.2 ng/mL), then the tacrolimus evening dose was held, and random tacrolimus was then monitored 24 hours after the last recent dose. The dose was still supratherapeutic (8 ng/mL), so the doses were held for 24 hours, and the tacrolimus level was repeated the next day. The level decreased to 4.4 ng/mL. Then, the oral dose of 1 mg tacrolimus was resumed every 12 hours. Furthermore, the *CYP3A5* genetic polymorphism was also investigated to explain why the trough levels were too high. It was reported that patients had poor metabolism of the *CYP3A5* enzyme (CYP3A5^*^3/^*^3 genotype), which showed that patients might require a lower dose of the target concentration of tacrolimus. In clinical setting of this case, tacrolimus trough levels were monitored daily because fluctuations in tacrolimus exposure are persistent during this period and due to hemodynamic instability, ileus, and liver function impairment with reduced cytochrome activity. Then, the doses were carefully adjusted to maintain therapeutic tacrolimus levels during the early period of OLT.

The relationships between tacrolimus dose, trough concentration, and relevant laboratory results are indicated in Table 1. The data in Table 2 were analyzed to determine the relationships among tacrolimus dose, trough concentration, significant clinical factors, and tacrolimus clearance using a pharmacokinetic prediction equation based on a population pharmacokinetics study.

**Table 1:** Relationships among tacrolimus dose, trough concentrations, and relevant laboratory data

Postoperative day	Tacrolimus dose	Tacrolimus trough concentration	Hb	Hct	AST	ALT	ALP	GGT	TBIL	DB	ALB	BUN	sCr	PT	INR
Reference ranges		Target 7–5	12–16	36–48	5–34	30–65	40–150	9–36	0–1	0–0.3	34–50	7–18	0.51–1	10.5–13.5	0.91–1.17
Unit	mg/day	ng/mL	g/dL	Percentage (%)	U/L				mg/dL		g/L	mg/dL			
D 0	*NA*	*NA*	7.9	23.2	9274	2546	70	106	9.6	6.2	22.4	18	1.11	32	2.88
POD 1	*NA*	*NA*	8.3	23.9	10,261	3107	117	220	10.4	6.3	35.9	47	2.45	29.2	2.61
POD 2	*NA*	*NA*	7.1	20.5	3488	1940	127	289	11.5	8.1	36.8	74	3.33	26.6	2.36
POD 3	2	3.5	7.8	22.1	2210	1566	137	321	11.7	8.5	32.8	92	4.15	50.4	4.69
POD 4	3	**8.1**	9.4	27	1123	1156	152	372	10.9	8.3	31.6	104	4.48	70.3	6.69
POD 5	3.5	**8.5**	8.9	25.6	457	642	120	277	7.8	6.2	24.5	86	3.38	66.8	6.33
POD 6	1	**9.1**	10.8	31.3	380	663	145	335	9.6	7.6	26.8	106	3.53	62.1	5.86
POD 7	1.5	**7.2**	9.9	28.2	205	467	125	296	8.1	6.5	25.6	101	2.94	49.9	4.64
POD 8	1	6.4	10.1	28.6	137	352	130	293	6.4	5.2	26.3	100	2.55	32	2.88
POD 9	1.5	6.7	10.3	29.8	99	284	142	340	6.2	4.8	25.2	85	2.29	20.8	1.82
POD 10	2.5	5.4	9.8	28.1	67	197	132	289	5.3	4.2	23.6	70	2.05	16.9	1.46
POD 11	6	**7.9**	NA	NA	57	166	147	303	5	3.9	23	64	1.86	*NA*	*NA*
POD 12	7	**9.1**	8.8	25.7	49	134	142	274	4.6	3.5	22.2	61	1.88	14.9	1.27
POD 13	7	**9.2**	8.2	23.8	46	115	138	253	4.4	3.4	21.9	59	2.07	*NA*	*NA*
POD 14	5.5	**9.4**	6.8	19.9	31	66	94	142	3.4	2.7	28	66	2.79	17.1	1.47
POD 15	2.5	**12.2**	8.6	25.5	32	68	103	147	3.4	2.6	28.9	72	2.85	14.6	1.24
POD 16	1/hold p.m. dose	**8**	8.7	25.8	27	58	107	138	3	2.3	30.5	66	2.48	15.5	1.33
POD 17	Hold 2 doses	4.4	8.4	25	27	46	109	133	2.7	2	31.2	55	1.73	15.1	1.29
POD 18	2	3.7	8.2	24.4	27	34	110	117	2.5	1.7	31	38	1.18	14.5	1.24
POD 19	5	5	8.3	24.7	25	30	110	112	2.4	1.6	26.3	32	0.95	14.4	1.23
POD 20	6	**7.7**	8.3	24.4	29	28	117	113	2.2	1.5	25.3	29	1.09	*NA*	*NA*
POD 21	5.5	5.6	7.6	22.5	30	27	115	110	2.2	1.5	26.1	26	0.93	15.1	1.29
POD 22	5.5	7.6	9.3	27.6	33	30	127	106	2.3	1.6	25	28	0.9	*NA*	*NA*

Bold values indicate the supratherapeutic tacrolimus trough concentrations.

*Hb* hemoglobin, *Hct* hematocrit, *AST* aspartate aminotransferase, *ALT* alanine transaminase, *ALP* alkaline phosphatase, *GGT* gamma-glutamyl transferase, *TBIL* total bilirubin, *DB* direct bilirubin, *ALB* albumin, *BUN* blood urea nitrogen, *sCr* serum creatinine, *PT* prothrombin time, *INR* international normalize ration, *NA* values not available, *D 0* operation day, *POD* postoperative day, *hold 2 doses*, omit both morning and evening dose of tacrolimus

**Table 2:** Relationships between tacrolimus dose, trough concentration, clinical factors, and tacrolimus clearance

Postoperative day	Tacrolimus dose	Tacrolimus trough concentrations	Hb	TBIL	Tacrolimus oral clearance (CL/F) calculated from the Eq. 26.2 × (Hb/11) ^−0.802^ × (TBIL/1.9) ^−0.096^
Reference ranges		Target 5–7	12–16	0–1	
Units	mg/day	ng/mL	g/dL	mg/dL	L/h
POD 3	2	**3.5**	7.8	11.7	28.99
POD 4	3	**8.1**	9.4	10.9	25.13
POD 5	3.5	**8.5**	8.9	7.8	27.12
POD 6	1	**9.1**	10.8	9.6	22.76
POD 7	1.5	**7.2**	9.9	8.1	24.81
POD 8	1	6.4	10.1	6.4	24.97
POD 9	1.5	6.7	10.3	6.2	24.65
POD 10	2.5	5.4	9.8	5.3	26.05
POD 11	6	**7.9**	*NA*	5	*NA*
POD 12	7	**9.1**	8.8	4.6	28.79
POD 13	7	**9.2**	8.2	4.4	30.59
POD 14	5.5	**9.4**	6.8	3.4	36.44
POD 15	2.5	**12.2**	8.6	3.4	30.18
POD 16	1/hold p.m. dose	**8**	8.7	3	30.27
POD 17	Hold 2 doses	4.4	8.4	2.7	31.45
POD 18	2	3.7	8.2	2.5	32.30
POD 19	5	5	8.3	2.4	32.11
POD 20	6	**7.7**	8.3	2.2	32.38
POD 21	5.5	5.6	7.6	2.2	34.75
POD 22	5.5	7.6	9.3	2.3	29.43

*Bold values indicate the supratherapeutic tacrolimus trough concentrations*

*Hb* hemoglobin, *TBIL* total bilirubin, POD, postoperation day, hold 2 doses omit both morning and evening dose of tacrolimus

*NA* not available

## Discussion

Tacrolimus dosage adjustment for maintaining optimal therapeutic tacrolimus concentrations was generally difficult in this patient because the trough of whole blood tacrolimus concentrations fluctuated in the early postoperative LT. In addition, the TDM routinely performed in practice cannot provide the optimal trough concentrations every time. It was hypothesized that fluctuations in tacrolimus levels may be associated with the coexistence of clinical factors. Thus, to help clinicians explain the variability of tacrolimus levels, minimize interindividual variability, and eventually improve patient outcomes, a population pharmacokinetic equation for Thai LT patients was used to predict the oral clearance (CL/F) of tacrolimus. The equation was “CL/F (unit: L/hour) = 26.2 × (hemoglobin (Hb)/11)^−0.802^ × (total bilirubin (TBIL)/1.9)^−0.096’’^ [8], which showed the relationship between Hb and TBIL levels and tacrolimus CL/F. According to the equation, Hb is the most influential clinical factor affecting the CL/F of patients treated with tacrolimus. This could be explained by the extensive distribution of tacrolimus in red blood cells. As the Hb concentration decreases, the free drug concentration in the plasma increases, resulting in increased CL/F [8]. A reduction in the whole blood concentration of tacrolimus was observed. The other significant factor was TBIL, which was negatively related to the CL/F of tacrolimus. This indicated that tacrolimus is primarily excreted via bile. Thus, increasing TBIL levels could reflect a decrease in bile excretion, which might affect tacrolimus elimination [8, 9].

According to Table 2, there was a relationship between tacrolimus dose, trough concentration, influential clinical factors, and oral tacrolimus clearance. At POD 15, the trough tacrolimus level (12.2 ng/mL) was highly supratherapeutic. However, the dose was reduced since the previous day. Increasing Hb might decrease the CL/F of tacrolimus and increase the tacrolimus level at POD 15. In this patient, the Hb level was the lowest at POD 14 because he developed acute dyspnea with acute anemia and massive ascites. Then, the patient was sent for investigation via CT of the whole abdomen due to suspected intra-abdominal bleeding. He was transferred to the ICU for intensive monitoring and bleeding treatment with blood transfusion. The population pharmacokinetics equation was applied in this case to explain the relationship between tacrolimus oral clearance and clinical factors resulting in tacrolimus levels. It could be strongly suggested that Hb and TBIL are two significant factors that should be considered concomitantly for adjusting tacrolimus dosing. The tacrolimus trough concentrations were analyzed using Statistical Package for the Social Sciences (SPSS version 26) to determine the correlation between tacrolimus trough concentrations and the CL/F of tacrolimus calculated from the equation. The results showed that the CL/F of tacrolimus was not significantly negatively correlated with the trough tacrolimus concentration (*r* = −0.09, *r*^2^ = 0.0081, *p* = 0.713; Pearson’s correlation). Albumin is another clinical factor that might affect tacrolimus concentration because of the high protein binding affinity of tacrolimus. However, the serum ALB concentration was not included in the prediction equation, because it was evaluated as an insignificant covariate in a recent population pharmacokinetics study of LT patients [8]. This may be explained by the presence of unbound tacrolimus or metabolites with a lower affinity for albumin, particularly in the early period after transplant [10], and tacrolimus could also bind to other proteins, such as α1-glycoprotein and lipoproteins [11, 12].

This case report aims to introduce how the pharmacokinetic equation could be applied clinically to optimize the critical drug dose of tacrolimus in liver transplant patient. It suggested that clinicians predict the trend of clearance-affected tacrolimus levels by estimating the trend of Hb and TBIL levels day by day for dose adjustment based on the predictive pharmacokinetic equation. It would be better to optimize the tacrolimus dose when integrating the significant variables that affect tacrolimus levels into consideration with a routine tacrolimus level monitoring in the immediate postoperative period, which is the most challenging period in liver transplantation.

## Conclusion

Hemoglobin and total bilirubin were significant clinical factors influencing the oral clearance of tacrolimus early after liver transplantation. A decrease in the HB concentration would increase the free drug concentration and therefore increase the CL/F. An elevated TBIL decreases the biliary excretion of tacrolimus, resulting in a decrease in the CL/F of tacrolimus.

## Data Availability

Available from corresponding author on reasonable request.
